# Impact of Climate Change and Human Activities on Suitable Distribution of *Rhodiola* Species in the Qinghai‐Tibet Plateau: Modeling Insights for Conservation Prioritization

**DOI:** 10.1002/ece3.72896

**Published:** 2026-01-07

**Authors:** Xiao‐xue Li, Bo Liu, Lu Wang, Jing‐kai Zhang, Ao‐jie Zuo, Xiu‐Ming Li, Yang‐Jing Peng, Kun Jin, Ai‐Li Qin

**Affiliations:** ^1^ Ecology and Nature Conservation Institute, Chinese Academy of Forestry Beijing China; ^2^ Key Laboratory of Biodiversity Conservation of National Forestry and Grassland Administration Beijing China; ^3^ Research Institute of Natural Protected Area, Chinese Academy of Forestry Beijing China; ^4^ College of Life and Environmental Sciences, Minzu University of China Beijing China; ^5^ Research Institute of Forestry, Chinese Academy of Forestry Beijing China; ^6^ East China Academy of Inventory and Planning of National Forestry and Grassland Administration Hangzhou Zhejiang China

**Keywords:** anthropogenic pressure, climate vulnerability, habitat suitability, MaxEnt, Qinghai‐Tibet plateau, *Rhodiola*

## Abstract

Using the MaxEnt model with climatic, topographical, soil, and human activity factors, this study predicted suitable habitats for eight *Rhodiola* species in the Qinghai‐Tibet Plateau (QTP) and analyzed conservation gaps via ArcGIS overlay analysis. Models demonstrated high accuracy, with area under the receiver operating characteristic curve (AUC) values ranging from 0.88 to 0.99. Human activities dominated habitat suitability for most species (contribution: 37.0%–76.4%), except *R. atsaensis* (RA), driven by climate (38.9%) and topography (32.8%). Current suitable habitats varied widely, with RA occupying the largest area (1.69 × 10^6^ km^2^), and *R. sacra* (RS) the smallest (5.61 × 10^4^ km^2^). Future climate scenarios show seven *Rhodiola* species (except RS) will expand, and all have increasing highly suitable areas. *R. smithii* and *R. tibetica* expand most; RS only expands under SSP1‐2.6 in 2090. Current nature reserve coverage protects 33.42% of the suitable habitats for *Rhodiola* species on the plateau, with national reserves accounting for 28.13% and other protected areas (PAs) only 5.29%. Protection efficiency varies significantly among species. RA has the highest protection rate (35.38%), while *R. bupleuroides* and RS show the lowest (~20%). National reserves exhibit protection rates of 13.11%–29.98% for suitable habitats, surpassing other‐level reserves (2.1%–8.27%). Conservation gaps are concentrated in ecologically sensitive zones such as the Hotan‐Ngari, Lhasa, and eastern Chamdo. Strikingly, protection of high and medium habitats remains extremely low (5.12%). The findings provide critical insights for prioritizing strategic conservation efforts and optimizing PA networks across the QTP, thereby addressing the current protection gaps and enhancing ecological connectivity.

## Graphical abstract

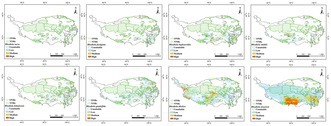

## 1 Introduction

Climate change and human activities, as dual threats, have triggered an accelerating decline in global biodiversity, pushing numerous species to the brink of extinction and disrupting ecosystem functions (Boonman et al. 2024; Ceballos et al. 2015). This trend is particularly evident on the Qinghai‐Tibet Plateau (QTP)—a globally significant biodiversity hotspot (Myers et al. 2000) and climate change‐sensitive region—where near‐future climate change may threaten high‐altitude endemics by drastically altering their distributions (Yan and Tang 2019). The plateau is experiencing a faster rate of warming compared to the Northern Hemisphere average (IPCC 2023), evidenced by altered precipitation patterns and intensified extreme weather events. Concurrently, human activities such as overgrazing, infrastructure expansion, and unregulated resource exploitation further exacerbate ecological stress.

Among the vulnerable flora of the QTP, the genus *Rhodiola*—a taxon of great ecological, medicinal, and conservation significance—comprises approximately 58 morphologically diverse species (Thiede and Eggli 2007) and has its origin and diversification center in the Tibetan Plateau and adjacent mountain regions (Zhang, Meng, Allen, et al. 2014; Zhang, Meng, Wen, et al. 2014). This genus represents a classic example of adaptive radiation in alpine plants. Its evolutionary history of diversification and the development of cold‐adaptation mechanisms in response to major geological events such as the uplift of the QTP make it an ideal model system for studying how montane herbaceous plants respond to environmental and climatic changes (You et al. 2018). However, the genus is now facing increasing extinction risks. As a pioneer alpine species valued for its key medicinal properties such as antihypoxia and antifatigue effects, *Rhodiola* is under severe threat from escalating market demand, anthropogenic activities, and climate change (Tao et al. 2019). Climate change, characterized by global warming, has led to rising temperatures, altered precipitation patterns, and frequent extreme climate events, significantly altering the distribution and quality of suitable habitats for *Rhodiola* species. Studies indicate that rising temperatures may force alpine plants to migrate to higher altitudes or cooler areas (Jia et al. 2015; Liang et al. 2015; Luo et al. 2016). Such changes also exert substantial impacts on the regional natural environment, ecosystems, and species distribution, further endangering threatened wild medicinal plant resources, including *Rhodiola* (Allen and Lendemer 2016; Fitzpatrick et al. 2008). Meanwhile, pervasive human activities particularly tourism infrastructure development and road construction on the plateau have directly led to habitat fragmentation and loss as quantified by recent studies (Jiang et al. 2023). Its high conservation value has led to the inclusion of the entire genus *Rhodiola* in CITES (the Convention on International Trade in Endangered Species of Wild Fauna and Flora, http://www.cites.org.cn/zxgg/zxzn/202404/t20240419_774718.html) Appendix II, underscoring its conservation urgency. Under the dual pressures of climate change and anthropogenic impacts (Zhang et al. 2015; Recio et al. 2016; Tao et al. 2019), *Rhodiola* has become a conservation priority, and our research is directly relevant to the development of proactive protection strategies. Therefore, it is imperative to model its distribution areas to clarify current and potential distribution patterns, thereby providing a scientific basis for effective conservation.

Furthermore, to ensure the reliability of our models, we built a comprehensive occurrence database by integrating high‐precision GPS locations from 2 years of systematic field surveys with carefully verified specimen records from the National Plant Specimen Resource Center (NPSRC) and the Global Biodiversity Information Facility (GBIF). This multisource data integration strategy significantly enhances the spatial and temporal coverage of our analysis, providing a solid foundation for robust distribution modeling. On the other hand, evaluating the effectiveness of the existing nature reserve (NR) system in protecting *Rhodiola* habitats is equally crucial. Although numerous NRs have been established on the QTP and have contributed to the conservation of the regional ecological environment and biodiversity, these reserves were predominantly planned around macroecosystems or flagship species, lacking targeted protection for economically and medicinally valuable plant groups like *Rhodiola*. Under the backdrop of climate change, NRs also face new challenges in species conservation. Therefore, it is particularly important to systematically assess the current reserve system's conservation efficacy for *Rhodiola* and to identify protection gaps.

Species distribution modeling (SDM) is a crucial tool for simulating suitable habitats and predicting distribution changes by integrating environmental variables and species distribution points (Huang et al. 2020; Dong et al. 2023). Among numerous species distribution models, the Maximum Entropy model (MaxEnt) has proven to be a superior methodology in academic research, owing to its simple operation, efficient computation, and consistent high prediction accuracy even with sparse data (Elith et al. 2011; Xu et al. 2019). Moreover, this model is also proficient in predicting the suitable habitats of endangered, medicinal, and alpine plants, and assessing their responses to climate change (Jia et al. 2022; Yang et al. 2024; Zhang et al. 2025).

## 2 Material and Methods

### 2.1 Study Area

**FIGURE 1:**
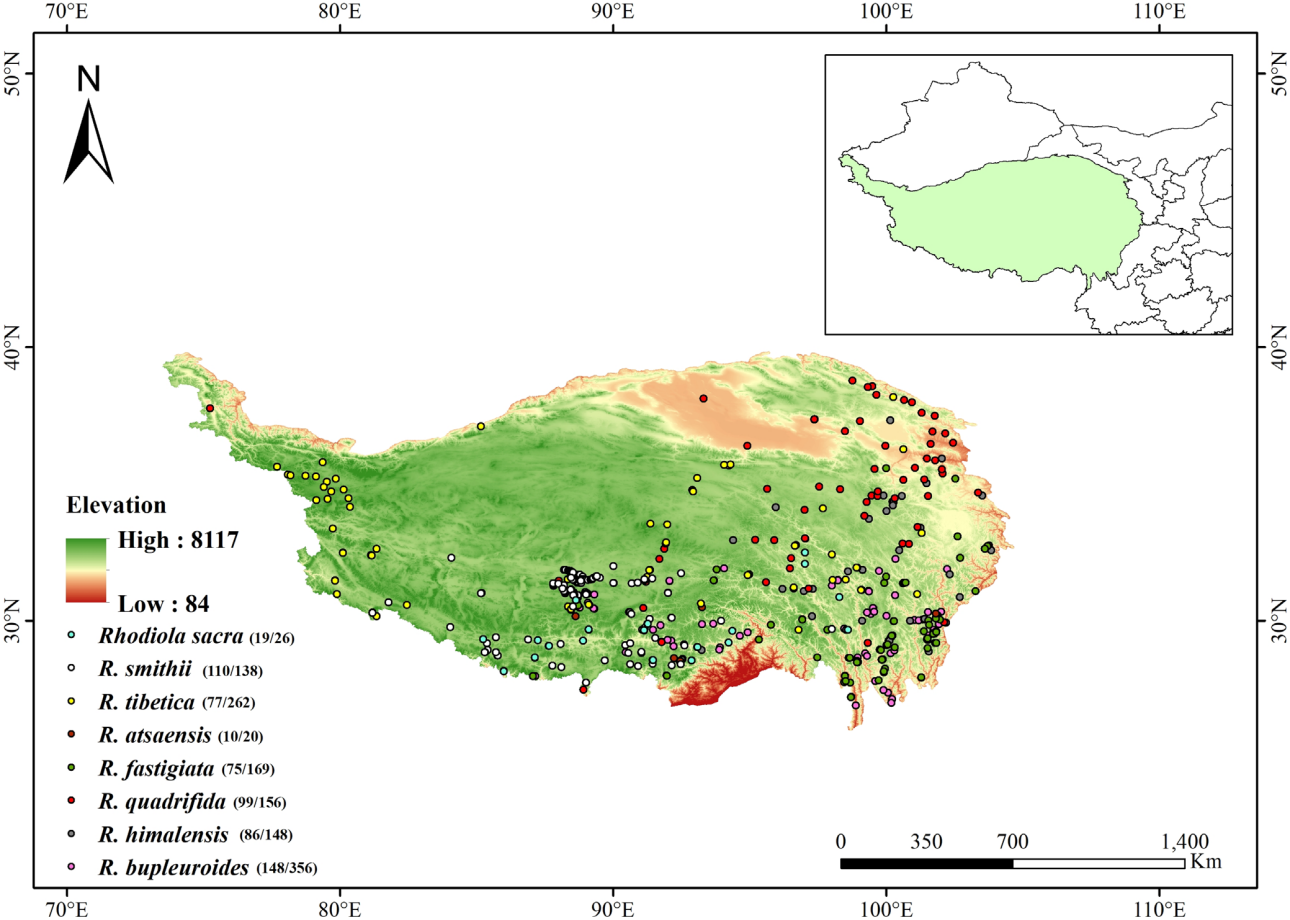
Distribution locations of eight *Rhodiola* species on the QTP. Numbers before and after the dash denote each species' prescreening and postscreening distribution points.

The QTP, an inland plateau in Asia, extends between latitudes 26°00′12″ N‐39°46′50″ N and longitudes 73°18′52″ E‐104°46′59″ E (Figure 1). Spanning 2.5 million square kilometers with an average altitude of 4500 m (Zhang 2019), the plateau boasts diverse and complex landscapes, including numerous mountain ranges and hydrological systems. Topographically, its elevation decreases from the northwest to the southeast. In terms of precipitation, there is a significant increase from the arid northwest to the more humid southeast. As for temperature, it shows a gradual decline from the south to the northwest (Dong et al. 2023).

### 2.2 Species Occurrence Data

The distribution data of eight *Rhodiola* species were sourced from two methods: (1) Collection of field investigation records from 2023 to 2024. (2) Retrieval from open‐source databases, namely the GBIF (available at https://www.gbif.org/) and the NPSRC (accessible at https://www.cvh.ac.cn). In total, 1275 pieces of data on the occurrence of eight *Rhodiola* species on the QTP were collected. Subsequently, these distribution data were filtered with ArcGIS 10.2 to ensure that each 1 × 1 km grid cell contained only one record of a single species. Eventually, a total of 624 occurrences of the eight *Rhodiola* species were obtained. The number of distribution points for each species before and after filtering is shown in Figure 1. Photographs of six of the eight species and their habitats are presented in Figure 9, the remaining two are excluded due to substandard field photographs.

### 2.3 Environmental Data

We chose 41 environmental variables to model the possibility distributions of eight *Rhodiola* species on the QTP (Figure S1), comprising 19 bioclimatic factors, 3 topographical ones, 17 variables related to soil, and 2 items associated with human activities. More precisely, 19 bioclimatic variables between 1950 and 2000 on the QTP were retrieved from the Global Climate Database (https://www.worldclim.org). Climate data in the 2050s (2041–2060) and 2090s (2081–2100) were also employed. The SSP scenarios, more specifically, SSP 1‐2.6 and SSP 5‐8.5, were utilized for predicting future climate conditions (Su et al. 2021; Chen and Yu 2025). The three topographical factors were derived as follows: elevation data was obtained from the Geospatial Data Cloud (https://www.gscloud.cn), and subsequently, slope and aspect were computed using ArcGIS 10.2. The 17 soil variables and two human‐activity factors were all acquired from the National Tibetan Plateau Science Data Center (TPDC, https://data.tpdc.ac.cn/home). The two human activity factors, the Human Footprint Index (HFI) and livestock density, were selected as they represent the most pervasive and direct anthropogenic pressures on the QTP ecosystem (Duan and Luo 2021). The HFI, which integrates multidimensional data on population density, land use, nighttime lights, and road/railway networks (Duan and Luo 2021; Luo et al. 2018, 2020), serves as a comprehensive metric of cumulative human disturbance. With values ranging from 0 to 50, it effectively captures the habitat fragmentation and cumulative disturbance intensity resulting from tourism development, infrastructure expansion, and resource collection, which represent the primary threats to medicinal plant resources on the QTP. Livestock grazing, primarily by yaks and sheep, is the most widespread land‐use practice on the QTP, directly impacting alpine meadow ecosystems through trampling and foraging, which alters habitat suitability for perennial herbs like *Rhodiola* (Mipam et al. 2019). All the above 41 environmental factors have a spatial resolution of 30 arc‐seconds, which is equivalent to approximately 1 km. The collinearity among numerous factors may cause the model to be overfitted, thereby having an impact on the prediction results (Hu 2014). To alleviate factor collinearity, Pearson's correlation analysis was performed using ArcGIS 10.2 (Figure S1). Variables with a Pearson's correlation coefficient |*r*| > 0.8 were removed (Yang et al. 2023). Eventually, 7 climatic, 3 topographic, 13 soil, and 2 human‐activity variables were selected for subsequent model simulations (Table 1). Finally, we used the ArcGIS 10.2 resampling tool to resample the resolution of the selected 16 environmental variable layers to 1 km for model analysis.

**TABLE 1:** The average AUC values and standard deviation across various time periods.

Abbreviated	Scientific name	LIG	Current	2041–2060 (2050s)		2081–2100 (2090s)	
				SSP1‐2.6	SSP5‐8.5	SSP1‐2.6	SSP5‐8.5
RS	*R. sacra*	0.976 ± 0.0296	0.980 ± 0.0202	0.977 ± 0.0238	0.984 ± 0.0136	0.986 ± 0.0141	0.984 ± 0.0168
RF	*R. fastigiata*	0.974 ± 0.0154	0.972 ± 0.0178	0.972 ± 0.0149	0.969 ± 0.0153	0.970 ± 0.0167	0.967 ± 0.0183
RQ	*R. quadrifida*	0.943 ± 0.0236	0.928 ± 0.0322	0.914 ± 0.0339	0.917 ± 0.0307	0.917 ± 0.0318	0.916 ± 0.0305
RB	*R. bupleuroides*	0.970 ± 0.0102	0.970 ± 0.0091	0.963 ± 0.0098	0.958 ± 0.0119	0.957 ± 0.0111	0.958 ± 0.0118
RH	*R. himalensis*	0.943 ± 0.0233	0.935 ± 0.0291	0.941 ± 0.0248	0.943 ± 0.0251	0.940 ± 0.0249	0.945 ± 0.0222
RT	*R. tibetica*	0.900 ± 0.0421	0.913 ± 0.0366	0.891 ± 0.0381	0.888 ± 0.0421	0.888 ± 0.0400	0.880 ± 0.0426
RA	*R. atsaensis*	0.881 ± 0.0907	0.947 ± 0.0297	0.929 ± 0.0529	0.916 ± 0.0434	0.920 ± 0.0705	0.901 ± 0.0429
RSM	*R. smithii*	0.981 ± 0.0072	0.980 ± 0.0077	0.972 ± 0.0106	0.974 ± 0.0091	0.974 ± 0.0085	0.974 ± 0.0107

### 2.4 Model Analyses and Evaluation

In this study, the MaxEnt model (version 3.4.4) was utilized to predict the potential habitats of eight *Rhodiola* species on the QTP. A total of 41 variables were initially considered. To mitigate overfitting and reduce model uncertainty, we conducted a multicollinearity test, retaining only variables with a Pearson's correlation coefficient |*r*| < 0.8 for final model calibration. To build and validate the model, we randomly designated 75% of the geographic distribution data as the training set and reserved 25% as the test set. For model evaluation and uncertainty quantification, we employed a bootstrap approach with 20 replicate runs, keeping other parameters at their default settings. This ensemble‐like process allowed us to generate not only a mean habitat suitability map but also a spatially explicit uncertainty map (measured as the standard deviation across replicates), which identifies regions where predictions are less reliable. The area under the receiver operating characteristic curve (AUC) for the averaged model and the average omission error were used to evaluate the accuracy of the model's performance (Phillips et al. 2006; Elith et al. 2011). A model's accuracy is judged as excellent (AUC 0.9–1), good (0.8–0.9), fair (0.7–0.8), poor (0.6–0.7), or failed (0.5–0.6) (Phillips et al. 2006). The final output of the mean suitability was logistic ASCII format, imported into ArcGIS 10.2 and converted to raster data. Using the “reclassify” tool in the spatial analysis module, the raster layers were divided into four habitat suitability levels: unsuitable habitat (0–0.1), low suitability habitat (0.1–0.4), medium suitability habitat (0.4–0.6), and high suitability habitat (0.6–1). The reclassified rasters were projected using the Albers Equal Area Conic projection (1000 × 1000 m pixel size), with pixel counts in medium‐ and high‐suitability classes representing their respective habitat areas.

### 2.5 Conservation Gaps Analysis for Plateau *Rhodiola* Species

To better understand the conservation status of the eight *Rhodiola* species on the plateau within the existing NR system, we used ArcGIS 10.2 software to conduct an overlay analysis of the medium and high suitable habitat of these species with the layers of national nature reserves (NNRs) and various types of NRs other than national nature reserves (ONRs) in the Tibetan Plateau region.

## 3 Results

### 3.1 Model Accuracy

For the current conditions and four future Shared Socioeconomic Pathways (SSPs) in the 2070s (2041–2060) and 2090s (2081–2100), the models showed AUC values ranging from 0.88 to 0.99 for all species, indicating good or excellent predictive accuracy (Table 1).

### 3.2 Key Environmental Variables

A critical finding from our MaxEnt modeling was the predominant role of anthropogenic pressure in shaping the suitable habitats of *Rhodiola* species. When variables were categorized into four types, human activity emerged as the most influential factor for the majority of the studied species (Table 2). This influence was particularly dominant for *R. sacra* (RS), *R. quadrifida* (RQ), and *R. himalensis* (RH), where the cumulative contribution of human activity variables exceeded 71.5%, surpassing all other factors. Similarly, for *R. fastigiate* (RF), *R. bupleuroides* (RB), *R. tibetica* (RT), and *R. smithii* (RSM), human activities remained the primary influencing factor, with contribution rates of 57.7%, 48.5%, 39.4%, and 37.0%, respectively. In contrast, *R. atsaensis* (RA) was an exception, with climate (38.9%) and topography (32.8%) being the major determinants, while human activity had a minimal impact (9.6%).

**TABLE 2:** The relative contributions (%) of variables to *Rhodiola* species by MaxEnt model.

Environmental variables (units)	Code	Contribution (%)							
		RS	RF	RQ	RB	RH	RT	RA	RSM
**Climate**		**19.1**	**12.3**	**11.5**	**29.5**	**8.5**	**26.2**	**38.9**	**21.9**
Mean diurnal range (°C)	Bio2	0.4	1	0.8	2.4	0.7	2.4	0	4.8
Isothermally	Bio3	**15.1**	0.5	1.4	**6.2**	0.6	1.4	**26.6**	0.3
Mean Temperature of Coldest Quarter (°C)	Bio11	0.3	1.2	0.9	**8.3**	0.5	4.4	1.5	4.5
Annual Precipitation (mm)	Bio12	0.7	**3**	0.6	3.4	**3.6**	3.3	0.7	**7.4**
Precipitation of Driest Month (mm)	Bio14	1.9	**3.3**	**4.1**	3.2	1.8	2.3	0.7	1.9
Precipitation Seasonality (mm)	Bio15	0.5	1.9	0.7	1.1	0.6	**6.6**	0.2	1
Precipitation of Coldest Quarter (mm)	Bio19	0.2	1.4	**3**	**4.9**	0.7	**5.8**	**9.2**	2
**Soil**		**5.4**	**26.1**	**7.6**	**14.7**	**11**	**19.9**	**18.8**	**34.4**
Silt (g/kg)	SLT	**2.7**	1.7	0.4	3.3	0.7	3.6	0.8	**17.5**
pH (H_2_O)	PH	0.9	**12**	1.3	2.7	**3.4**	2.2	**11**	0.5
Clay (g/kg)	CLY	0.7	1.9	0.7	0.4	1.2	0.6	0.5	0.4
Cation Exchange Capacity (cmol^(+)^/kg)	CEC	0.3	0.3	0.4	0.2	0.2	0.4	1.4	**11.9**
Soil Properties (%)	CF	0.3	2.1	0.8	2.8	0.2	5.6	0	0.6
Thickness (cm)	TCS	0.2	0.9	1.1	0.5	0.8	1.7	0	0.2
Total Phosphorus (g/kg)	TP	0.1	0.2	0.6	0.5	1.3	0.8	1	0.2
Soil Organic Carbon Density (kg/m^2^)	SOCD	0.1	0.3	0.2	0.8	0.4	1.3	0	1
Soil texture classes	STC	0.1	0.8	0.2	0.7	0.3	0.5	1.9	0.5
Total Potassium (g/kg)	TK	0	1.3	0.3	0.4	0.1	0.1	0.9	0.4
Total Potassium Density (kg/m^2^)	TKD	0	1.4	0.6	1.4	0.7	1.1	0.3	0.2
Bulk Density (g/cm^3^)	BD	0	0.8	0.6	0.5	1.5	1.6	0.9	0.7
Total Phosphorus Density (kg/m^2^)	TPD	0	2.4	0.4	0.5	0.2	0.4	0.1	0.3
**Topography**		**4.0**	**3.8**	**4.9**	**7.1**	**4.1**	**14.3**	**32.8**	**6.8**
Aspect	ASPE	**2.5**	1	1.1	1.1	**2.1**	3	1.8	1
Slope (°)	SLOP	1.4	0.5	1.4	1.4	0.4	1.4	2.9	1
Elevation (m)	ASL	0.1	2.3	**2.4**	4.6	1.6	**9.9**	**28.1**	4.8
**Human activity**		**71.5**	**57.7**	**76.0**	**48.5**	**76.4**	**39.4**	**9.6**	**37.0**
Human Footprint	HFP	**69.4**	**57.1**	**74.0**	**47.7**	**75.8**	**35.2**	3.9	**34.4**
Livestock carrying capacity (MU/km^2^)	LCC	2.1	0.6	2.0	0.8	0.6	4.2	5.7	2.6

*Note:* Bold values represent the cumulative contribution rate of each factor category (climate, soil, topography, human activity); colored values indicate the contribution rates of the top 4 most influential variables (for species suitable distribution) across all factors (regardless of category).

This pattern is further substantiated by the performance of individual variables. The HFI alone emerged as one of the most influential predictors, with remarkably high permutation importance. Specifically, the contribution of HFI exceeded 47% for five of the eight species and surpassed 69% for three species (RS, RQ, and RH). This consistently high contribution at the individual variable level underscores that human disturbance is a primary driver of current distribution patterns for these medicinal plants, often outweighing the influence of key climatic and edaphic factors for most species studied. A more in‐depth analysis of the top four variables for each species confirms this trend: for seven of the eight species, HFI was the single most substantial influencing factor (Table 2, Figure S2). The other critical factors varied by species: for instance, RS and RB were codominated by temperature‐associated variables (e.g., Isothermality, Bio3), while RQ and RT were strongly influenced by precipitation‐related variables (e.g., Precipitation of Driest Month, Bio14). The distribution of RF and RSM was notably driven by soil‐related factors, with the distribution of RSM being intricately regulated by the interaction between soil silt content and cation‐exchange capacity.

### 3.3 Potential Distribution of *Rhodiola* Species Under Current Climate Conditions

**FIGURE 2:**
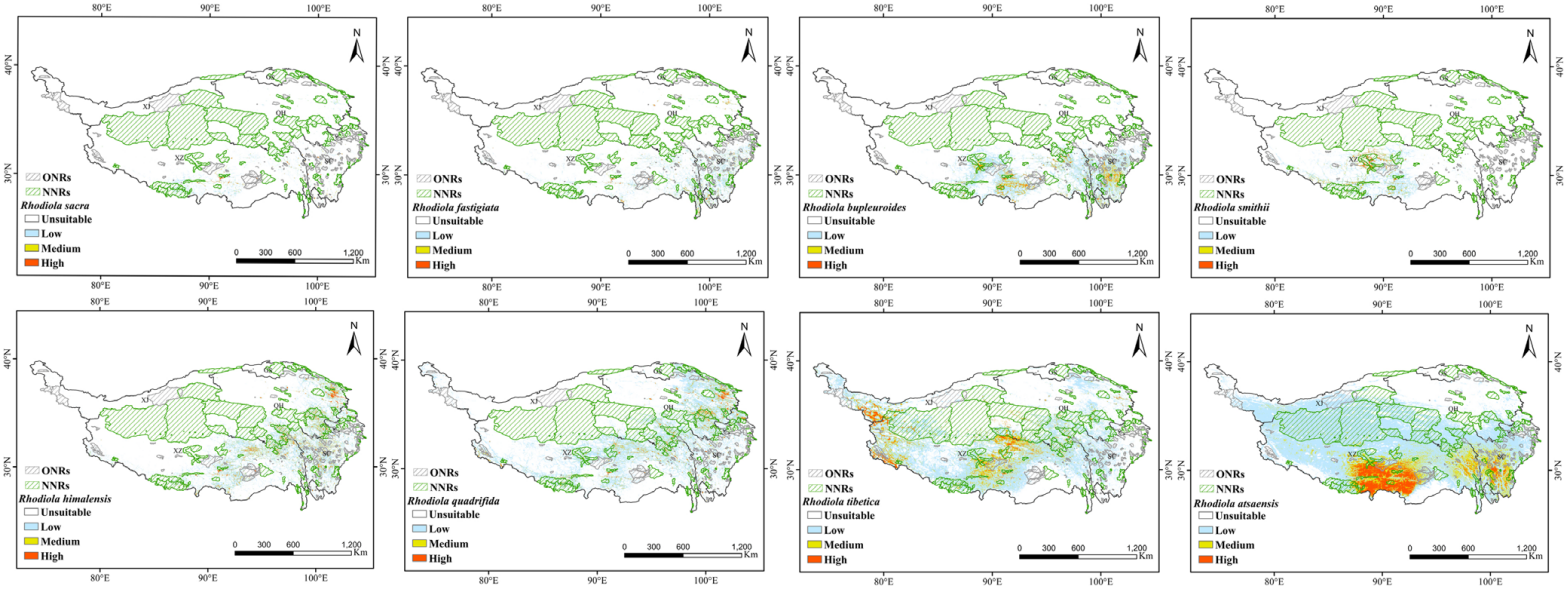
Current potential suitable areas for eight *Rhodiola* species.

The modeling results for the distribution ranges of eight *Rhodiola* species under the current climate conditions are provided in Figure 2, Table 3 and Table 4. Eight species of *Rhodiola* have distinct distribution patterns across the QTP. RA has the largest suitable habitat area (1.69 × 10^6^ km^2^), with 3.21 × 10^5^ km^2^ of high and medium suitability. Its habitats are concentrated in the southern and southeastern parts of QTP, including southern Nagqu, Lhasa, eastern Shigatse, northwestern Shannan, eastern Qamdo in Tibet, and Garze in Sichuan. Next is RT with a suitable habitat area of 1.02 × 10^6^ km^2^ among which high and medium suitability habitats cover 1.79 × 10^5^ km^2^. Its high and medium suitability habitats span across Hotan of Xinjiang, western Ngari, eastern Nagqu, Lhasa, northeastern and southern Shigatse in Tibet, and Yushu in Qinghai. RQ and RH rank third and fourth in terms of suitable distribution areas, which are 6.50 × 10^5^ km^2^ and 4.36 × 10^5^ km^2^ respectively, with high/medium suitability habitats covering 7.22 × 10^4^ km^2^ and 7.16 × 10^4^ km^2^. Their habitats nearly overlap in northeastern Nagqu, Lhasa, northern Qamdo in Tibet, southeastern Yushu, western Hainan, and Xining in Qinghai. The suitable distribution area of RB is 3.84 × 10^5^ km^2^, among which high and medium suitability habitats cover 6.83 × 10^4^ km^2^. RB is distributed in four main regions: southern Nagqu; the border area of Shannan, Nyingchi, and Lhasa; the Qamdo‐Yushu junction; and Ganzi in Sichuan. The remaining three species have a relatively small area of most suitable habitat. RSM has an area of 1.49 × 10^5^ km^2^ and occurs in small patches in southern Nagqu, central and southern Shigatse, Lhasa, and northern Shannan in Tibet. *R. fastigiata* (RF, 1.73 × 10^5^ km^2^) and RS (5, 6124 km^2^) are sporadically scattered in southern Lhasa, northern Shannan, northeastern Shigatse, and northwestern Nyingchi.

**TABLE 3:** The total and high suitable area of *Rhodiola* species under current and future climate scenarios.

Abbr.	Species	Current (km^2^)		2050 Total (km^2^)		2090 Total (km^2^)		2050 High (km^2^)		2090 High (km^2^)	
		Total	High	SSP1‐2.6	SSP5‐8.5	SSP1‐2.6	SSP5‐8.5	SSP1‐2.6	SSP5‐8.5	SSP1‐2.6	SSP5‐8.5
RS	*R. sacra*	56,124	2746	38,337	54,282	57,731	43,375	2761	4521	4956	3946
RF	*R. fastigiata*	173,296	7413	204,493	245,599	223,853	196,165	9215	12,048	11,319	8477
RQ	*R. quadrifida*	649,580	24,501	698,175	786,213	730,378	758,536	24,508	24,945	29,256	31,681
RB	*R. bupleuroides*	384,319	21,923	472,109	493,642	511,568	498,833	25,698	22,296	26,757	23,911
RH	*R. himalensis*	436,437	25,660	442,614	511,251	526,901	485,974	27,188	31,127	35,544	26,631
RT	*R. tibetica*	1,020,366	51,215	1,268,977	1,251,255	1,218,049	1,259,350	89,903	89,622	91,421	89,158
RA	*R. atsaensis*	1,688,933	125,700	2,346,513	2,118,547	2,102,316	2,113,896	299,969	251,244	138,378	146,295
RSM	*R. smithii*	149,304	11,253	211,624	212,504	218,193	195,774	24,294	26,142	25,676	24,498
All	All species	1,979,073	219,305	2,371,662	2,247,280	2,218,121	2,225,332	389,674	356,421	273,944	255,996

### 3.4 Suitable Habitat Fluctuations in the Future

Predictions from future climate models suggest that under the SSP1‐2.6 and SSP5‐8.5 scenarios for 2050, the total suitable distribution areas of plateau *Rhodiola* species are 2.37 × 10^6^ km^2^ and 2.25 × 10^6^ km^2^, respectively, with their highly suitable areas measuring 3.90 × 10^5^ km^2^ and 3.56 × 10^5^ km^2^. For 2090, these projections show total suitable areas of 2.22 × 10^6^ km^2^ and 2.23 × 10^6^ km^2^ under the same scenarios, accompanied by highly suitable areas of 2.74 × 10^5^ km^2^ and 2.56 × 10^5^ km^2^, respectively (Table 3). Compared with the present, the future potential suitable distribution of plateau *Rhodiola* species as a whole shows an expansion trend.

**FIGURE 3:**
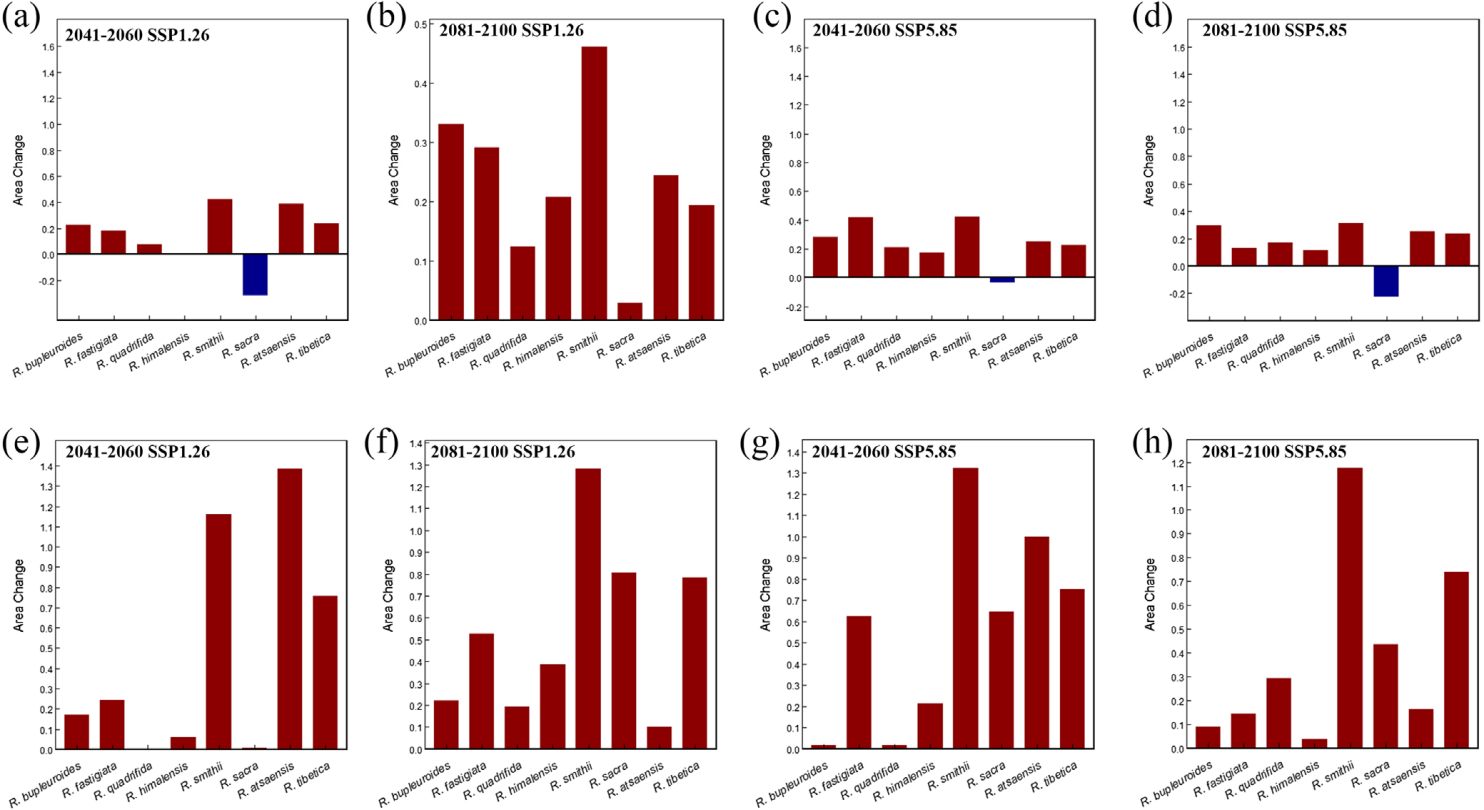
Changes in the area of total and high suitable habitats for eight plateau *Rhodiola* species under different future climate scenario models. (a–d) Total suitable distribution area; (e–h) Highly suitable distribution area.

**FIGURE 4:**
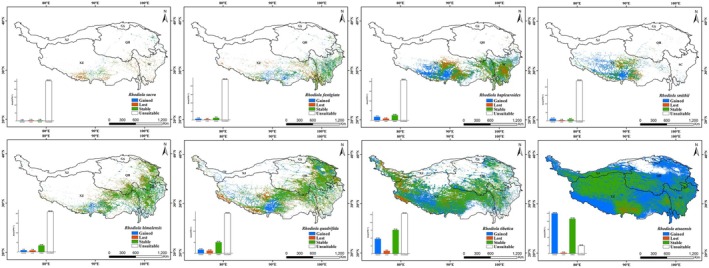
Changes in the potential suitable distributions of eight plateau *Rhodiola* species under SSP1‐2.6 scenario in 2050.

**FIGURE 5:**
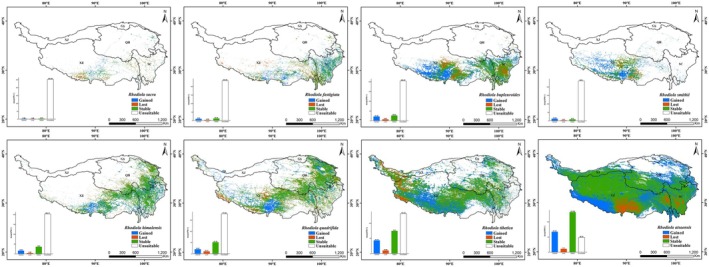
Changes in the potential suitable distributions of eight plateau *Rhodiola* species under SSP5‐8.5 scenario in 2050.

**FIGURE 6:**
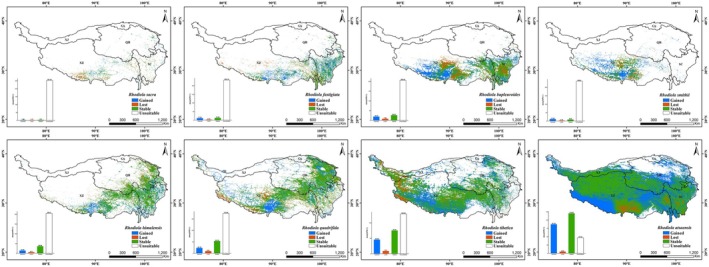
Changes in the potential suitable distributions of eight plateau *Rhodiola* species under SSP1‐2.6 scenario in 2090.

**FIGURE 7:**
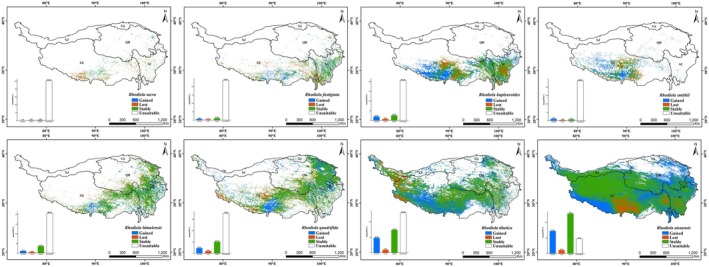
Changes in the potential suitable distributions of eight plateau *Rhodiola* species under SSP5‐8.5 scenario in 2090.

For each species, under the SSP1‐2.6 and SSP5‐8.5 scenarios for 2050 and 2090, seven of the eight plateau *Rhodiola* species showed expanding potential suitable areas. Only RS exhibited spatiotemporal variation: its suitable area expanded under SSP1‐2.6 in 2090 but contracted under SSP1‐2.6/SSP5‐8.5 in 2050 and SSP5‐8.5 in 2090 (Table 3 and Figure 3). The highly suitable distribution areas of all eight species showed an expansion trend (Table 3, Figure 3). Under the 2050 SSP1‐2.6 and SSP5‐8.5 scenarios, RA, RSM, and RT exhibited the most substantial expansions in their highly suitable habitats. Specifically, these species showed 1.4‐fold, 1.2‐fold, and 0.8‐fold increases under SSP1‐2.6, and 1‐fold, 1.3‐fold, and 0.8‐fold increases under SSP5‐8.5. In contrast, RQ and RS displayed minimal growth under SSP1‐2.6, with virtually no change in area, while RQ and RB showed only 444 and 373 km^2^ expansions (0.02‐fold) under SSP5‐8.5. For the 2090 SSP1‐2.6 and SSP5‐8.5 scenarios, RSM and RT continued to lead in expansion, with maximal increases of approximately 1.3‐fold and 0.8‐fold, respectively. RS showed significant growth of 0.8‐fold under SSP1‐2.6 and 0.4‐fold under SSP5‐8.5, whereas RA and RH exhibited the most minimal expansion—10% and 4%, respectively—under their corresponding scenarios. The suitable distribution areas of these species are projected to expand outward from their current locations, as illustrated in Figures 4, 5, 6, 7 and Figures S3–S6.

### 3.5 The Conservation Status of Eight Plateau *Rhodiola* Species Under the Existing Protected Area (PA) Systems

**FIGURE 8:**
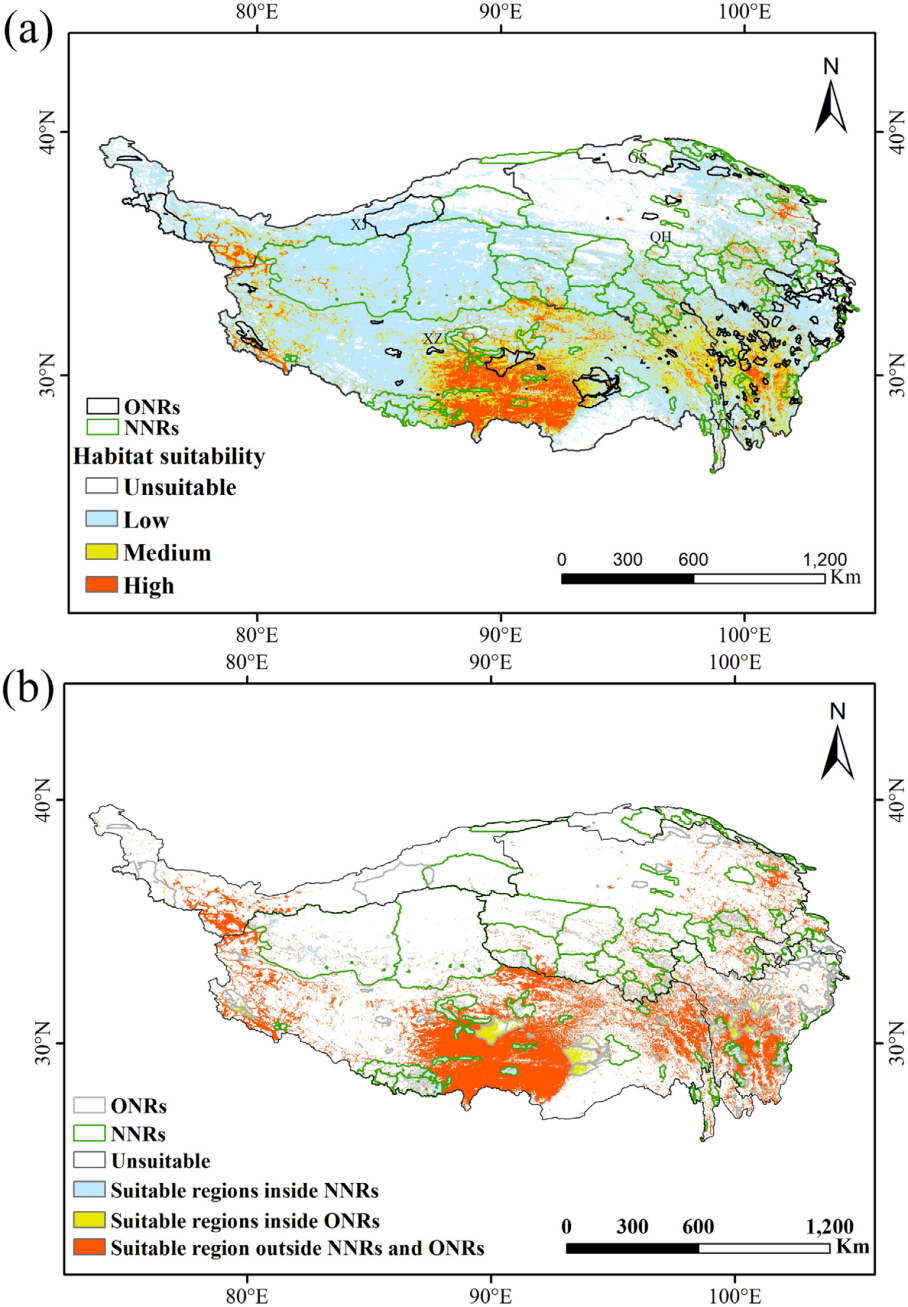
Overlay analysis of potential suitable distribution of plateau *Rhodiola* species (a) and protection gaps for their medium‐ and high‐suitability habitats under current reserves (b).

Overlay analysis of the eight *Rhodiola* species' potential habitats and current reserves shows plateau *Rhodiola* face significant protection challenges in the current NR system (Figure 8a, Table 4). NNRs on the QTP protected 1, 979, 073 km^2^ of *Rhodiola* suitable habitats (28.13% of the total), with other nature PAs encompass 104, 687 km^2^ (5.29%). Together, the existing PA system on the QTP conserves 33.42% of the plateau's *Rhodiola* suitable habitat, while the protection rate for their high and medium habitats is only 5.12% (Table 4). Conservation gap analysis of high and medium suitable habitats with the existing PA system shows that protection deficits are primarily concentrated in the border region of Hotan (Xinjiang) and Ngari (Tibet), specifically the western fringe of Qiangtang National Nature Reserve; the regions surrounding Lhasa, eastern Shigatse, and northwestern Shannan precisely correspond to the vicinities of four NNRs: the southern sector of Serling Tso National Nature Reserve, the areas encircling Lalu Wetland and the Yarlung Zangbo River Middle Reaches Valley Black‐necked Crane National Nature Reserves, and the eastern portion of Qomolangma National Nature Reserve; and eastern Chamdo in Tibet (Figure 8b). For each species, the existing PA system shows the highest protection rate for RA (35.38%), while RB and RS have the lowest rates (both ~20%) (Table 4). Moreover, the protection efficiency of suitable habitats for the eight *Rhodiola* species varies significantly between national and other‐level NRs: national reserves exhibit rates from 13.11% (RF) to 29.98% (RA), whereas other‐level reserves show rates of 2.1% (RS) to 8.27% (RF) (Table 4).

**TABLE 4:** Area and proportion of high, medium, and low suitable habitats for *Rhodiola* species protected by national nature reserves (NNRs) and other nature reserves (ONRs) under the current climate scenario.

Abbr.	Scientific name	Classes	Suitable Area	Protected by NNRs		Protected by ONRs	
				Area (km^2^)	Proportion (%)	Area (km^2^)	Proportion (%)
RS	*R. sacra*	High	2746	121	0.22	21	0.04
		Medium	4077	442	0.79	105	0.19
		Low	49,301	9584	17.08	1051	1.87
		Total	56,124	10,147	18.08	1177	2.10
RF	*R. fastigiata*	High	7413	750	0.43	319	0.18
		Medium	12,858	1593	0.92	875	0.50
		Low	153,025	20,378	11.76	13,141	7.58
		Total	173,296	22,721	13.11	14,335	8.27
RQ	*R. quadrifida*	High	24,501	4112	0.63	516	0.08
		Medium	47,716	9911	1.53	1381	0.21
		Low	577,363	105,769	16.28	23,333	3.59
		Total	649,580	119,792	18.44	25,230	3.88
RB	*R. bupleuroides*	High	21,923	2970	0.77	1252	0.33
		Medium	46,459	5755	1.50	3329	0.87
		Low	315,937	39,200	10.20	24,442	6.36
		Total	384,319	47,925	12.47	29,023	7.55
RH	*R. himalensis*	High	25,660	3627	0.83	1188	0.27
		Medium	45,984	7985	1.83	2326	0.53
		Low	364,793	56,152	12.87	20,660	4.73
		Total	436,437	67,764	15.53	24,174	5.54
RT	*R. tibetica*	High	51,215	6830	0.67	2101	0.21
		Medium	127,360	23,552	2.31	5126	0.50
		Low	841,791	184,637	18.10	32,823	3.22
		Total	1,020,366	215,019	21.07	40,050	3.93
RA	*R. atsaensis*	High	125,700	5196	0.31	6331	0.37
		Medium	195,578	21,101	1.25	17,991	1.07
		Low	1,367,655	480,081	28.43	66,774	3.95
		Total	1,688,933	506,378	29.98	91,096	5.39
RSM	*R. smithii*	High	11,253	3817	2.56	275	0.18
		Medium	16,050	4016	2.69	523	0.35
		Low	122,001	20,131	13.48	4393	2.94
		Total	149,304	27,964	18.73	5191	3.48
All	All species	High	219,305	21,142	1.07	10,067	0.51
		Medium	318,037	48,612	2.46	21,547	1.09
		Low	1,441,731	487,030	24.61	73,073	3.69
		Total	1,979,073	556,784	28.13	104,687	5.29

## 4 Discussion

### 4.1 Distribution of *Rhodiola* and Fluctuations of Its Habitat Under Ecological and Human Factors

Integrating the Maxent model and GIS, we predicted the potential distributions of eight *Rhodiola* species on the QTP and characterized their spatial patterns and habitat suitability. AUC values ranged from 0.88 to 0.99 for all species, demonstrating high accuracy in the models.

Current suitable habitat models showed that moderately and highly suitable habitats for *Rhodiola* species were primarily located in the Himalayas of the QTP, certain central plateau areas, and the Hengduan Mountains (HMs). This discovery corresponds with previous research by Zhang, Meng, Allen, et al. (2014); Zhang, Meng, Wen, et al. (2014), which identified the QTP and its adjacent mountainous areas as the origin and diversification center of the *Rhodiola* genus. Additionally, our simulation results for RF are consistent with earlier molecular phylogeographic outcomes (Zhang et al. 2018), which demonstrated that the QTP and the HMs acted as crucial refugia, from which *Rhodiola* lineages spread and occupied wider geographical areas. Our simulation results also largely concur with previous models by Yang et al. (2023) and You et al. (2018), which solely incorporated climatic factors for *Rhodiola* species RB, RF, RH, RQ, and RS. Both sets of findings identified the eastern and southern QTP, along with the HMs, as suitable habitats for *Rhodiola*. Significantly, our integrated model, which accounts for topographical, soil, climatic variables, and human activities, projects a smaller extent of suitable habitats compared to previous climate‐only simulations (Yang et al. 2023; You et al. 2018).

Under climate change, suitable habitats for seven of eight QTP *Rhodiola* species are projected to expand, with RS showing contrasting trends: expansion under 2090 SSP1‐2.6, but contraction under 2050 SSP1‐2.6/SSP5‐8.5 and 2090 SSP5‐8.5. This aligns with You et al. (2018), who found most of 14 *Rhodiola* species expanded under 2050 RCP8.5 (single climatic factor), except *R. alsia* and RQ. Further corroborating these findings, Yang et al. (2023) simulated four *Rhodiola* species under the RCP8.5 scenario for 2050 and 2070 using a single climatic factor. They observed a significant increase in the suitable habitat area of *R. coccinea* in both 2050 and 2070, while RQ also exhibited an expansion pattern in 2070. *R. integrifolia* in North America also demonstrated a comparable range‐extension pattern (Forester et al. 2013), providing additional evidence for the widespread distribution expansion trend across the *Rhodiola* genus. In the QTP ecosystem, three *Hippophae* species also expanded their ranges under climate warming (Jia et al. 2015). These results contradict the “nowhere to go” hypothesis (Loarie et al. 2009; Nogués‐Bravo et al. 2007), shedding light on alpine plants' responses to climate change and enhancing our understanding of alpine flora dynamics. Several factors can account for this expansion pattern. First, these species are generally restricted to small areas in the eastern and southeastern QTP, leaving extensive high‐altitude regions in the north and west unexplored, providing ample space for their range expansion. Second, climate warming‐induced increases in temperature and precipitation have the potential to convert previously inhospitable areas, such as permafrost regions, into suitable habitats at higher latitudes and altitudes. Third, human activities play an indirect role. Ecological conservation initiatives enhance the overall environment, while transportation infrastructure development creates marginal habitats and promotes seed dispersal, contributing to the expansion of *Rhodiola* species' distribution.

### 4.2 Human Activities as the Primary Driver of Plateau *Rhodiola* Habitat

Climate change and increasing anthropogenic activities have exerted substantial impacts on the vegetation of the QTP (Zhu et al. 2022). While earlier modeling of species habitats relied predominantly on climatic variables (Yang et al. 2023; You et al. 2018), recent studies emphasize the critical importance of integrating soil, topography, and human‐activity data to improve prediction accuracy (Yang et al. 2022; Dong et al. 2023). Our study reveals a decisive and somewhat unexpected finding: contemporary human activities exert a pressure on the distribution of alpine Rhodiola species that is at least comparable to, and for most species far exceeds, that of climate. Specifically, human activities were identified as the main factor affecting the distribution of second‐class protected plants RS, RQ, RF, and RH, with contribution rates of 71.5%, 76%, 57.7%, and 76.4%, respectively. This result significantly diverges from prior investigations by You et al. (2018) and Yang et al. (2023), which, being limited to climatic factors, concluded that temperature‐related variables were the key regulators. The immense contribution of the HFI in our models suggests that infrastructure, tourism, and associated disturbances are not merely secondary factors but are primary forces currently redefining the conservation landscape for these vulnerable plants. This finding aligns with a growing body of literature documenting the severe impacts of anthropogenic pressure on fragile alpine ecosystems, where the rapid expansion of tourism infrastructure and road networks fragments habitats and increases accessibility for illegal harvesting, posing an immediate threat that may eclipse the more gradual effects of climate change in the short term (Chen et al. 2014; Zhu et al. 2022).

Human activities also emerged as the primary influencing factors for RB, RT, and RSM, with contribution rates of 48.5%, 39.4%, and 37.0%, respectively. The highest contribution rates underscore that land use, infrastructure construction, medicinal resources collection, and tourism have profoundly affected the growth, reproduction, and distribution of plateau species (Xu et al. 2019). Consequently, our results advocate for a strategic shift in conservation planning—from a primary focus on climate impacts to a dual‐focused approach that confronts immediate, localized human threats with the same rigor as global climate change.

Beyond human activities, climate, topography, and soil factors also play important but species‐specific roles. Precipitation of the Driest Month (Bio14) is key for RQ, while Annual Precipitation (Bio12) is critical for RH. Isothermality (Bio3) and Mean Temperature of the Coldest Quarter (Bio11) respectively dominate RS and RB. Elevation is crucial for RT and the less human‐affected RA, while soil properties such as pH and silt content are decisive for RF and RSM. This not only indicates the crucial impact of human activities in determining species distribution but also implies each species' unique ecological needs and adaptation strategies. This pattern not only confirms the overarching impact of human activities but also reflects each species' unique ecological niche and adaptation strategy, which must be carefully considered in cultivation and conservation programs.

### 4.3 Climate and Topography as the Primary Driver of RA Habitat

**FIGURE 9:**
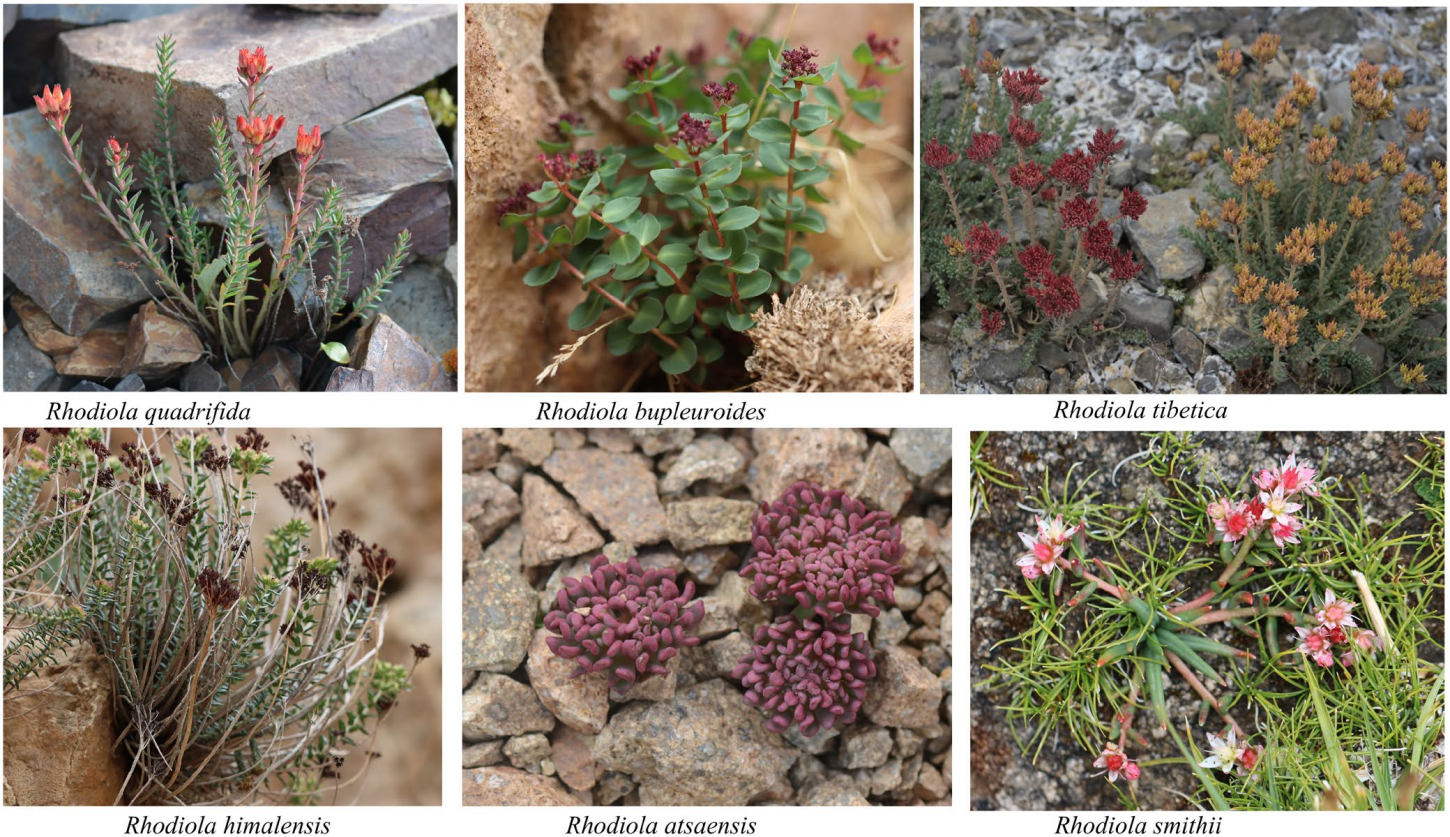
Photographs of six representative *Rhodiola* species and their habitats. Photos by: Bo Liu ( *R. quadrifida* , *R. atsaensis*); Yuantong Hou (*R. bupleuroides*, *R. himalensis*); Xinxin Zhou (*Rhodiola tibetica*); Aili Qin (R. *smithii* ).

A notable exception among the eight plateau *Rhodiola* species is RA, for which human activities exert only a negligible impact (9.6%). In contrast, its suitable distribution is predominantly shaped by temperature and topographic factors, which collectively account for 71.7% of the model contribution. This demonstrates its distinctive environmental niche—being more sensitive to abiotic conditions and less affected by anthropogenic disturbance compared to its congeners. We attribute this divergence to the species' specialized microhabitat preference; field surveys confirm that RA is a specialist of high‐alpine scree slopes (Figure 9). These landforms are intrinsically governed by microclimate and terrain, while their unsuitability for grazing and farming inherently buffers them from major human impact. Consequently, the species' survival is closely linked to its physical environment, whereas the broader‐ranging species exhibit stronger anthropogenic signals due to greater overlap with human activity zones.

### 4.4 Specialist‐Climate Versus Generalist‐Human‐Driven Distributions

Our analysis of the eight *Rhodiola* species reveals that their distribution drivers can be categorized into two archetypal patterns based on niche width: climate‐sensitive specialists and human‐impacted generalists. Specialist species with narrow ecological amplitudes (e.g., RA) primarily inhabit stable microhabitats dominated by abiotic factors, such as high‐altitude scree slopes. Their distributions are closely linked to specific hydrothermal conditions and topographic features, and their occurrence in remote areas minimizes direct human disturbance. In contrast, generalist species with broader niche widths occupy more productive and accessible habitats (e.g., alpine meadows). Their widespread distribution leads to extensive overlap with human activities (e.g., grazing, cultivation), resulting in population dynamics that are primarily driven by anthropogenic pressures. This central finding not only explains the divergent distribution patterns observed in our study but also provides a predictive framework for assessing regional biotic vulnerability. Under ongoing environmental change, we project that these two groups will face distinct threats: specialists risk the disruption of their abiotic niches, while generalists are more susceptible to direct habitat loss and degradation.

### 4.5 Identifying Conservation Gaps and Optimizing Conservation Frameworks on the QTP

Previous research by Guo et al. (2025) has demonstrated that NRs are the most effective means of wildlife conservation and biodiversity preservation in China. Our research on *Rhodiola* species, however, reveals a critical nuance in this effectiveness. While the current PA system on the QTP covers 33.42% of the total suitable habitat for *Rhodiola*—a proportion higher than the 16.7% protection coverage reported for plateau endemic species by Yan and Tang (2019). However, the core issue is not merely the presence of protection gaps, but their specific location in high‐ and medium‐suitability zones, such as the Xinjiang‐Tibet border, Lhasa, eastern Shigatse, northwestern Shannan, and eastern Chamdo. This spatial mismatch reveals an imbalance between area coverage and ecological representativeness, highlighting a critical limitation of area‐based conservation targets: they can be met without adequately protecting core habitats.

This discrepancy stems from two main issues. Firstly, the existing PA network suffers from a significant “conservation bias”: its historical planning prioritized iconic ecosystems and flagship animals (Su et al. 2019), inadvertently neglecting endangered plants like *Rhodiola*. Consequently, protection gaps are predominantly concentrated in the interstitial spaces between different PAs. Furthermore, the static nature of past planning failed to incorporate the dynamic impacts of climate change and human disturbance, lacking the foresight to adapt to shifting species distributions.

Therefore, our study demonstrates that the success of the “30 × 30” framework and similar goals must be measured not just by the total area protected, but equally by the quality of ecological representation. To address these challenges and optimize the PA system, we propose a two‐pronged strategy: First, transition from a static to a dynamic and integrated conservation framework. It is imperative to establish a “plants‐animals‐ecosystems” integrated protection framework, granting endangered plants equal priority to animals and ecosystems. Given that the major protection gaps are clustered around existing reserves, the most efficient approach would be to strategically expand these reserves or establish new plant‐specific protection zones in these high‐priority gaps, thereby weaving a more comprehensive and resilient protection network. Second, leverage ecological modeling for proactive and adaptive conservation planning. When optimizing PAs, employing tools like the MaxEnt model and GIS technology is crucial. These methods allow conservationists to account for the impacts of climate change and human activities. By Runnig multiscenario simulations, we can identify not only current but also future potential habitats, enabling the proactive adjustment of reserve boundaries to keep pace with habitat evolution. This data‐driven approach ensures that the PA system of tomorrow is both representative and resilient, integrating broad quantitative targets with the qualitative prioritization of irreplaceable, high‐value habitats.

## 5 Limitations and Future Perspectives

First, a primary source of uncertainty in our future projections stems from the use of climate data from a single general circulation model (GCM), BCC‐CSM2‐MR. While this approach provides an internally consistent narrative of potential climate impacts under two contrasting emission scenarios (SSP1‐2.6 and SSP5‐8.5), it does not capture the full range of intermodel variability inherent in climate projections. Different GCMs can simulate divergent future climates, particularly for complex topographic regions like the QTP. Consequently, our habitat suitability maps for the future represent one plausible trajectory based on the BCC‐CSM2‐MR model. To strengthen the robustness of such projections, future work should employ a multimodel ensemble approach. Analyzing the consensus and divergence across multiple GCMs would allow for the identification of high‐confidence priority areas and a more nuanced quantification of projection uncertainty. Despite this, the consistent habitat expansion trend observed across both contrasting emission scenarios within this model framework enhances our confidence in the general direction of change, even if the exact magnitude remains uncertain.

Second, while our model results benefit from extensive sampling across key environmental gradients and spatial filtering to reduce bias, several limitations merit discussion as they inform future research. A primary concern is the uneven sampling across certain elevational bands, which may affect the fine‐scale habitat suitability maps. Specifically, predictions above 5500 m should be interpreted as ecological inferences rather than empirical projections, given the absence of field validation in these extreme environments. To address this, future work should (1) integrate correlative models with process‐based physiological models to theoretically validate distribution limits, and (2) employ emerging technologies like environmental DNA (eDNA) or high‐resolution remote sensing for indirect validation in inaccessible areas. Despite these sampling challenges, our dataset represents one of the most comprehensive field compilations for *Rhodiola* species to date, providing a robust baseline for identifying major distribution patterns and protection gaps.

Another limitation pertains to the MaxEnt model's assumption that additive variable relationships may not fully capture nonlinear interactions between climate and human activity factors. Future work should prioritize interaction modeling frameworks such as Structural Equation Modeling, Generalized Additive Models, or Bayesian Additive Regression Trees to better elucidate complex driver mechanisms. Nevertheless, by establishing the significant independent roles of climate and human factors, our study lays a solid groundwork for future investigation into their interactive effects.

Finally, our static gap analysis mainly focused on the static distribution and protection status of habitats and did not incorporate dynamic landscape connectivity and habitat fragmentation effects. Future work should integrate landscape ecology approaches such as Landscape Graph Theory and Circuit Theory to model functional connectivity, identify key corridors and stepping stones, and ultimately enhance the resilience of conservation networks to climate and anthropogenic pressures. By explicitly accounting for both structural and functional connectivity, these methods would substantially improve conservation planning in this dynamic landscape. Our static analysis nonetheless provides an essential assessment for conservation prioritization, immediately highlighting regions where fundamental protection is most lacking.

## 6 Conclusions

This study employed the MaxEnt model and ArcGIS to map suitable habitats of eight *Rhodiola* species on the QTP, with high AUC values (0.88–0.99) validating the model's reliability. Key findings revealed that human activities were the primary driver of habitat distribution for most species, highlighting the urgent need to balance conservation with human development. Exceptionally, RA was more strongly influenced by climate and topography, indicating species‐specific ecological adaptability. Spatial analysis showed significant variation in habitat sizes, with RA occupying the largest area (1.69 × 10^6^ km^2^) and RS the smallest (5.61 × 10^4^ km^2^). Under future climate scenarios, seven of eight species will experience habitat expansion, yet the extent varies. RSM exhibits the most substantial growth potential, indicating strong resilience to climate change, while RS shows only slight expansion under the SSP5‐8.5 scenario in 2050, with contraction observed in all other temporal and climatic scenarios, signaling its potential vulnerability.

Despite habitat expansions of most *Rhodiola* species under climate change, their conservation remains critically inadequate, with only 33.42% of suitable habitats currently safeguarded. Major protection gaps have been identified in Hotan‐Ngari, Lhasa, and eastern Chamdo, demanding immediate intervention. To address these gaps and ensure the long‐term persistence of *Rhodiola* species, we propose the following targeted conservation strategies: First, for the Hotan‐Ngari Region located in the western part of the Qiangtang National Nature Reserve and spanning the border between the Tibet Autonomous Region and Xinjiang, we recommend establishing cross‐regional collaborative management and incorporating this corridor into the “Qinghai‐Tibet Plateau National Park Cluster,” using our spatial data as a reference for boundary optimization. Second, for the southeastern Nagqu Region, situated at the junction of Lhasa and Xigazê and surrounded by major NRs (e.g., Selincuo, Mid‐reaches of Yarlung Zangbo River Black‐necked Crane Nature Reserve, Maidika Wetland) yet remaining a protection vacancy itself, we propose a twofold approach given its higher human population density: implementing a habitat quality improvement plan focusing on grassland restoration and regulating medicinal plant harvesting, while simultaneously piloting community‐based “protection cells” to empower local residents in sustainable monitoring and management. Third, for the eastern Chamdo corridors—the north–south valleys connecting established reserves like Gexigou, Haizishan, and Gongga Mountain, which serves as both a key connectivity pathway and a historically significant glacial refugia—we recommend formal designation as a priority climate adaptation corridor. Conservation efforts should emphasize habitat restoration and erosion control within these north–south valleys to maintain landscape connectivity under climate change. In summary, these measures will not only enhance the efficiency of the existing PA system but also provide a replicable model for alpine plant conservation in data‐constrained regions.

## Author Contributions

**Xiao‐xue Li:** investigation (equal), software (equal), validation (equal), visualization (equal), writing – original draft (equal). **Bo Liu:** investigation (equal). **Lu Wang:** investigation (equal). **Jing‐kai Zhang:** investigation (equal). **Ao‐jie Zuo:** software (supporting). **Xiu‐Ming Li:** investigation (equal). **Yang‐Jing Peng:** software (supporting). **Kun Jin:** conceptualization (lead). **Ai‐Li Qin:** conceptualization (lead), investigation (lead), resources (equal), supervision (equal), writing – review and editing (lead).

## Funding

This work was supported by the 2024 Survey Project of Rare and Protected Plants in Serling Tso National Nature Reserve from Naqu Forestry and Grassland Bureau.

## Conflicts of Interest

The authors declare no conflicts of interest.

## Supporting information


**Figure S1:** Correlation coefficient matrix of environmental variables.
**Figure S2:** Jackknife test of variable importance for eight plateau *Rhodiola* species.
**Figure S3:** Potential suitable areas for eight plateau *Rhodiola* species under SSP1‐2.6 scenario in 2050.
**Figure S4:** Potential suitable areas for eight plateau *Rhodiola* species under SSP5‐8.5 scenario in 2050.
**Figure S5:** Potential suitable areas for eight plateau *Rhodiola* species under SSP1‐2.6 scenario in 2090.
**Figure S6:** Potential suitable areas for eight plateau *Rhodiola* species under SSP5‐8.5 scenario in 2090.

## Data Availability

We utilized open‐access data from the Global Biodiversity Information Facility (GBIF, https://www.gbif.org/), the National Platform for Specimen Resources Collection (NPSRC, https://www.cvh.ac.cn), WorldClim (the Global Climate Database, https://www.worldclim.org), the Geospatial Data Cloud (https://www.gscloud.cn), and the Tibetan Plateau Data Center (TPDC, https://data.tpdc.ac.cn/home).
