# Supplementary material for: Impact of Climate Change and Human Activities on Suitable Distribution of Rhodiola Species in the Qinghai‐Tibet Plateau: Modeling Insights for Conservation Prioritization
Source: Ecol Evol. 2026 Jan 7;16(1):e72896. doi: 10.1002/ece3.72896 (PMC12778409; doi:10.1002/ece3.72896)

**Figure S1: Correlation coefficient matrix of environmental variables**


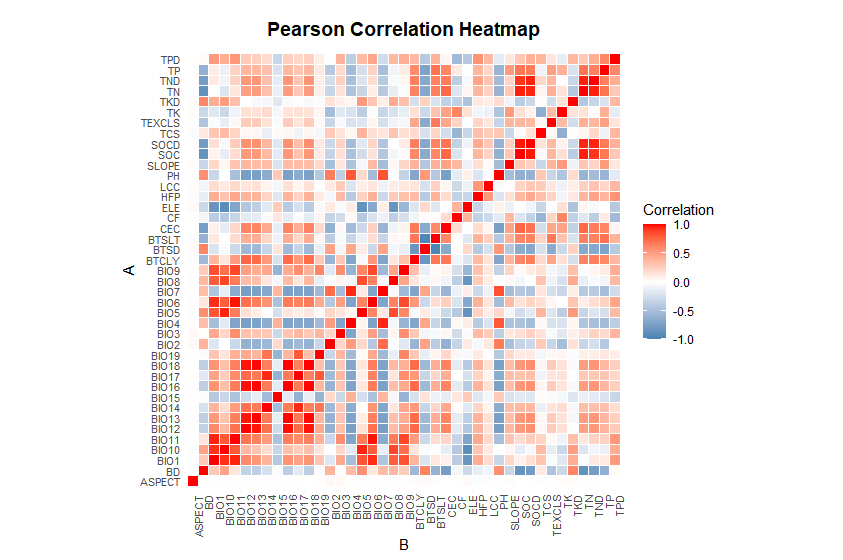


**Figure S2:** Jackknife test of variable importance for eight plateau *Rhodiola* species
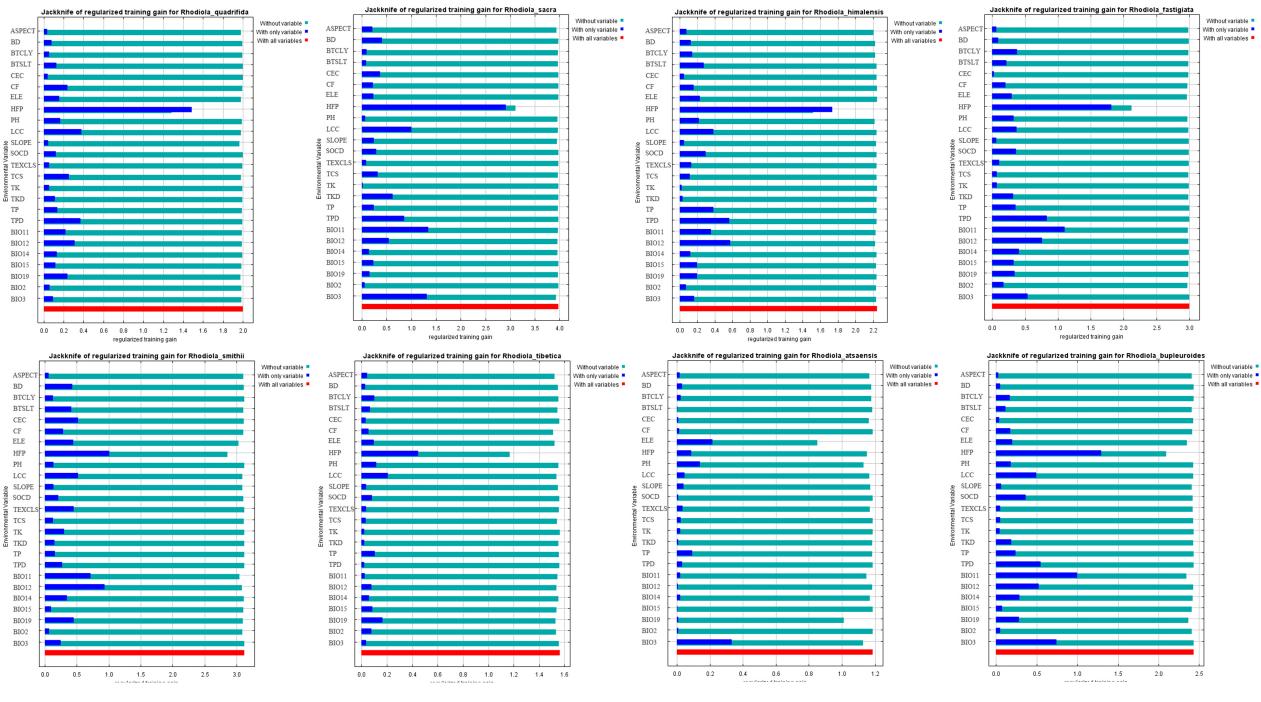


**Figure S3:** Potential suitable areas for eight plateau Rhodiola species under SSP1-2.6 scenario in 2050


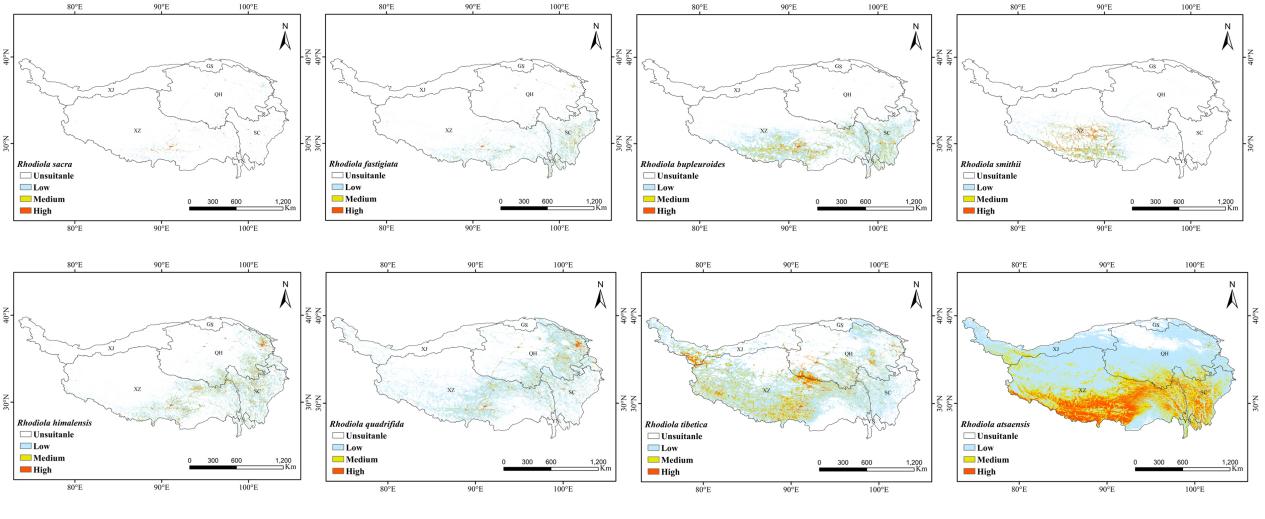


**Figure S4:** Potential suitable areas for eight plateau Rhodiola species under SSP5-8.5 scenario in 2050


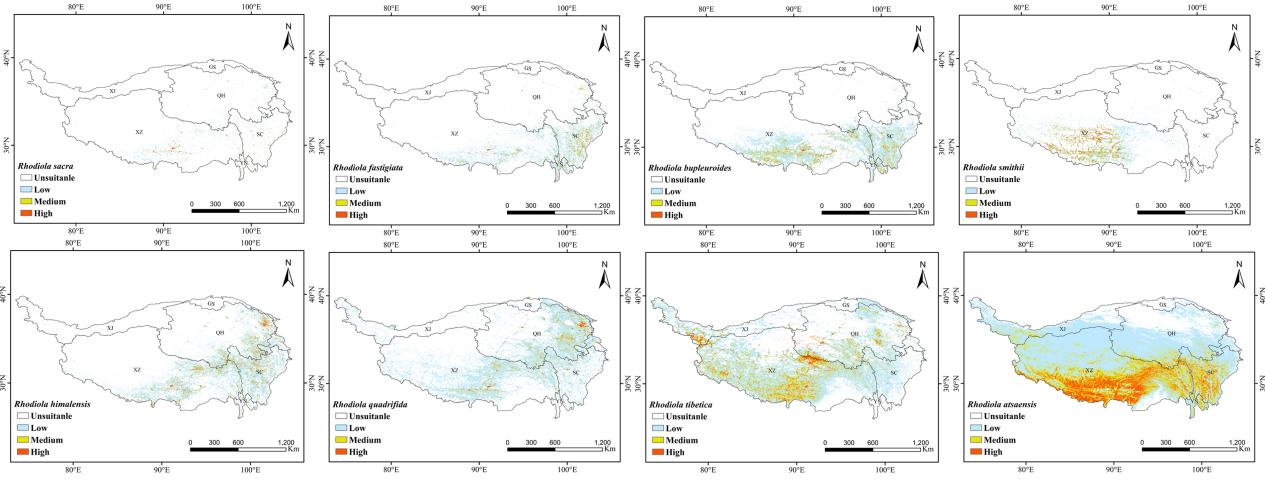


**Figure S5:** Potential suitable areas for eight plateau Rhodiola species under SSP1-2.6 scenario in 2090


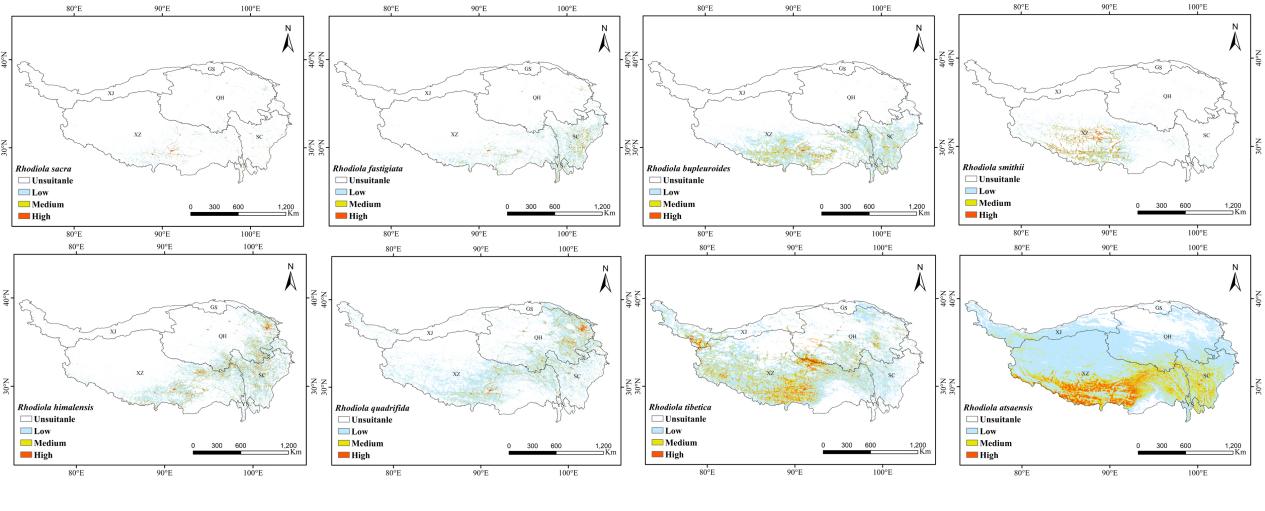


**Figure S6:** Potential suitable areas for eight plateau Rhodiola species under SSP5-8.5 scenario in 2090


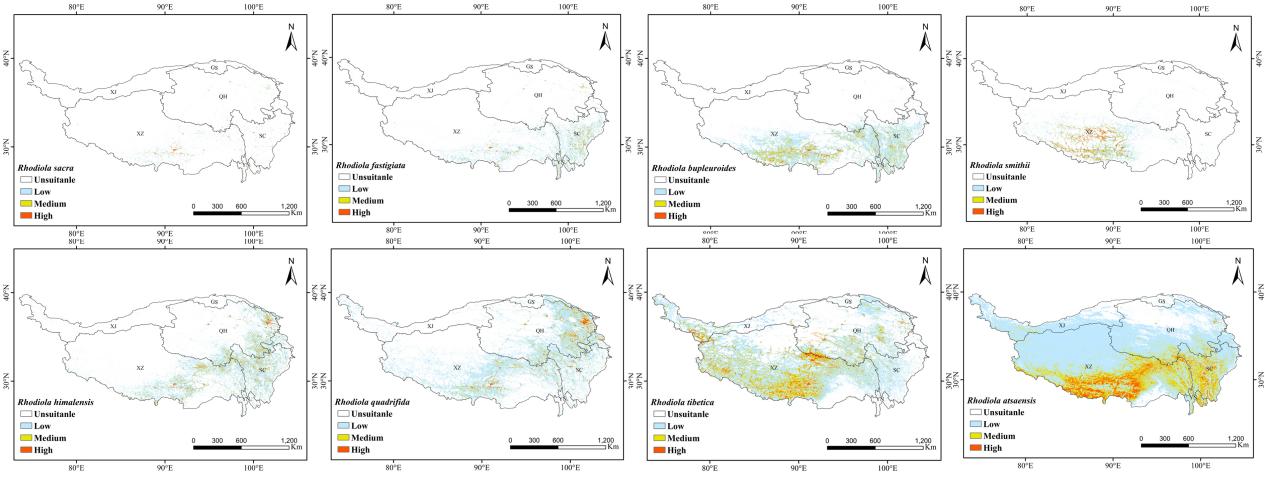

Supplement: Supplementary file 1 — Figure S1: Correlation coefficient matrix of environmental variables. Figure S2: Jackknife test of variable importance for eight plateau Rhodiola species. Figure S3: Potential suitable areas for eight plateau Rhodiola species under SSP1‐2.6 scenario in 2050. Figure S4: Potential suitable areas for eight plateau Rhodiola species under SSP5‐8.5 scenario in 2050. Figure S5: Potential suitable areas for eight plateau Rhodiola species under SSP1‐2.6 scenario in 2090. Figure S6: Potential suitable areas for eight plateau Rhodiola species under SSP5‐8.5 scenario in 2090. [file ECE3-16-e72896-s001.docx]
